# The effect of bisphosphonates on gene expression: GAPDH as a housekeeping or a new target gene?

**DOI:** 10.1186/1471-2407-6-49

**Published:** 2006-03-03

**Authors:** Maria Teresa Valenti, Francesco Bertoldo, Luca Dalle Carbonare, Giuseppe Azzarello, Sonia Zenari, Mirko Zanatta, Elena Balducci, Orazio Vinante, Vincenzo Lo Cascio

**Affiliations:** 1 Medicina Interna D, Department of Biomedical and Surgical Sciences, University of Verona, Italy; 2 Department of Oncology and Hematologic Oncology - Noale Hospital, Italy

## Abstract

**Background:**

RT-PCR has been widely used for the analysis of gene expression in many systems, including tumor samples. GAPDH (Glyceraldehyde-3-phosphate dehydrogenase) has been frequently considered as a constitutive housekeeping gene and used to normalize changes in specific gene expression. However, GAPDH has been shown to be up-regulated in many cancers and down-regulated by chemotherapic drugs. Bisphosphonates, potent inhibitors of bone resorption, have recently shown a direct and indirect antitumor effect in vitro and in animal models. They exert their effects mainly by inhibiting the mevalonate pathway but also by modulating the expression of many genes not only in osteoclasts but also in cancer cells.

**Methods:**

We evaluated GAPDH gene expression by real time RT PCR in breast (MCF-7 and T47D) and prostate (PC3 and DU-145) cancer cell lines treated with amino and non-amino bisphosphonates.

**Results:**

Our results showed that amino-bisphosphonates significantly decrease in a dose-dependent manner the expression of GAPDH gene.

**Conclusion:**

Therefore, GAPDH is inaccurate to normalize mRNA levels in studies investigating the effect of bisphosphonates on gene expression and it should be avoided. On the other hand, this gene could be considered a potential target to observe the effects of bisphosphonates on cancer cells.

## Background

GAPDH (Glyceraldehyde-3-phosphate dehydrogenase) is well known for its glycolytic function of converting D-Glyceraldeide-3-phosphate to 1,3-bisphosphoglycerate and it has been commonly considered as a constitutive housekeeping gene. It is widely used as a control RNA in Northern Blotting and in RT-PCR analysis and recently in real time RT-PCR.

In some experimental systems its expression is constant at different times and after experimental manipulation [1]. In breast cancer cells treated with endoxifen GAPDH was used to normalize the expression data of the progesterone receptor mRNA [2]. In addition, GAPDH was the best control gene in the apoptosis pattern on the myeloid cell lines incubated with Camptothecin investigated by real time RT-PCR [3].

However, there is overwhelming evidence suggesting that its use as an internal standard is inappropriate [4]. Growth hormone, oxidative stress and the tumour suppressor TP53 have all been shown to activate its transcription, which can also be induced in endothelial cells [5]. Conversely, retinoic acid down-regulates GAPDH transcription in adipocytes [6]. Furthermore, it has been observed that GAPDH mRNA expression was not normal in some tumour samples and its distribution exhibited a wide range of values. GAPDH mRNA was over-expressed in the poorly differentiated BT-20 cell line and the treatment of these cells with 1,25-dihydroxyvitamin D3 (1,25-(OH)_2_D3) stimulated GAPDH mRNA expression in a dose- and time-dependent manner [7]. A significant increase in GAPDH expression was observed when MCF-7 cells were stimulated with several factors as oestradiol, insulin growth factor 1 (IGF1) and basic fibroblast growth factor (bFGF) [8]. In addition, it has been observed that the GAPDH was up-regulated in rat hepatomas [9], malignant murine cell lines [10] and human prostate carcinoma [11].

GAPDH was also widely utilized as a control gene in studies conducted in the last decade to elucidate by RT-PCR the cellular effects of bisphosphonates, not only on osteoclasts or osteoblasts, but also on tumor cells [12,13].

Bisphosphonates (BPs), synthetic analogs of pyrophosphate, are potent inhibitors of bone resorption through the inhibition of osteoclast activity and recruitment [14,15]. They are used in many metabolic bone diseases. Furthermore, recent studies have demonstrated that BPs have an anti-tumour activity too, as highligthed by a reduced skeletal tumour burden and a slower progression of bone lesions in animal models [16]. BPs inhibit proliferation, cell adhesion to non-mineralised bone matrices and induce the apoptosis of a variety of human tumour cell lines in vitro [17-20]. Most of the BPs pharmacological activities have been related to inhibition of the mevalonate pathway [21], but the modulation of relative expression of a variety of genes implicated in osteoclast, osteoblast and tumour cell function has recently been reported [22-24]. On this basis and since the GAPDH is commonly used as housekeeping gene, also in studies using bisphosphonates, and since it is upregulated in many cancer (7–9,11) and downregulated by chemotherapic drugs (6), we assessed the effects, if any, of some bisphosphonates commonly used in cancer bone disease on GAPH gene expression in breast and prostate cancer cell lines.

## Methods

### Cells

Human prostatic cancer cell lines (PC-3 and DU-145) and human breast cancer cell lines (MCF-7 and T-47D) were purchased from the American Type Culture Collection (ATTC Rockville, MD, USA). The cells were maintained at 37°C in a humidified atmosphere with 5% CO_2 _in DMEM-F12 containing 2 mM L glutamine, 10% fetal bovine serum, 100 U/ml streptomycin and 100 μg/ml penicillin.

### Bisphosphonates studies

Alendronate (4-amino-1-hydroxybutilene-1,1-bisphosphonate), clodronate (tetrahydrate dichloromethylene-bisphosphonate, disodium), and pamidronate (3-amino-1-hydroxypropylidene-bisphosphonate acid, 2Na) were purchased from Calbiochem. Zoledronate (1-hydroxy-2,1-imidazol-1-yl-ethylidene bisphosphonic acid) was kindly provided by Novartis. The neutralized sodium salts of BPs were dissolved in sterile double distilled H_2_O at a final concentration of 100 mM. Stock solutions were aliquoted and kept at -20°C for long term storage.

Cells from 80% confluent cultures were washed with PBS and treated with trypsin/EDTA. Cells were plated at 1 × 10^6 ^in 25 cm^2 ^flask and incubated for 24 h at 37°C, allowing adhesion of the cells to the new culture plates. Cells were treated for 48 h with BPs concentrations of 100, 50, and 10 μM in DMEM/F12 containing 5% FBS. For each concentration three separate flasks were treated.

### Total RNA extraction

Before RNA extraction apoptotic cells were cut out by harvesting medium for each fask. Adherent cells were washed twice with PBS and trypsinized; the cell pellets were collected by centrifugation at 1000 g for 10 min at 4°C. Total RNA was extracted from each cell culture flask using the RNAeasy minikit (Quiagen) with DNAse I treatment. The amount of extracted RNA was quantified by measuring the absorbance at 260 nm. The purity of the RNA was checked by measuring the ratio of the absorbance at 260 and 280 nm, where a ratio ranging from 1.8–2.0 was taken to be pure. The absence of degradation of the RNA was confirmed by RNA electrophoresis on a 1.5% agarose gel containing ethidium bromide.

### Reverse transcription

First-strand cDNA was generated from 1 μg of each flask using the High-Capacity cDNA Archive Kit, with random hexamers, (Applied Biosystems PE) according to the manufacturer's protocol. RT product was aliquoted in equal volumes and stored at -80°C.

### Real time PCR

PCR was performed in a total volume of 50 μl containing 1x Taqman Universal PCR Master mix, no AmpErase UNG and 5 μl of cDNA; pre-designed, Gene-specific primers and probe sets for each gene (GAPDH; Hs99999905-m1) (Beta 2 microglobulin (B2M); Hs99999907) were obtained from Assay-on-Demand Gene Expression Products (Applied Biosystems). The real time amplifications included 10 minutes at 95°C (AmpliTaq Gold activation), followed by 40 cycles at 95°C for 15 seconds and at 60°C for 1 minute. As previously reported, the Ct value correlates to the starting quantity of target mRNA [25]. PCR efficiencies were calculated with a relative standard curve, derived from a four cDNA dilution series in triplicate and gave regression coefficients greater than 0.98 and efficiencies greater than 96%. To normalize the GAPDH mRNA expression from sample to sample in RNA input, quality and reverse transcriptase efficiency, we amplified the housekeeping gene B2M. The B2M endogenous/internal control gene was abundant and remained constant, in proportion to total RNA, among the samples. The GAPDH and B2M ratio represented the normalized GAPDH (the GAPDH/B2M ratio).

### Standard curves

The relative standard curves were obtained using the GAPDH and B2M gene primers and probes in singleplex, amplified with 10, 20, 40 and 80 ng of total RNA, respectively for each control cell line. Each sample was run in triplicate. The curves obtained for each cell line showed a linear relationship between RNA concentration and the Ct value of PCR real time for both GAPDH gene and B2M gene.

We selected the ΔRn in the exponential phase of amplification plots to determine the Ct values and to obtain the linearity of calibration curves.

### Statistical analysis

Results are expressed as mean ± S.E.M. Student's paired t-test was used to evaluate differences between the sample of interest and its respective control. The Wilcoxon test was used for nonparametric data. For analysis of dose responses, multiple measurement ANOVA followed by Newman-Keuls as a post-hoc analysis was performed. A probability value < 0.05 was considered statistically significant. Analyses were applied to experiments carried out at least three times. Statistical analyses were performed using Statgraphics Plus, version 5 (Manugistics, Inc, Rockville, MD).

## Results

Our results showed that BPs decrease, in a dose-dependent manner, the expression of the GAPDH gene.

**Figure 1:**
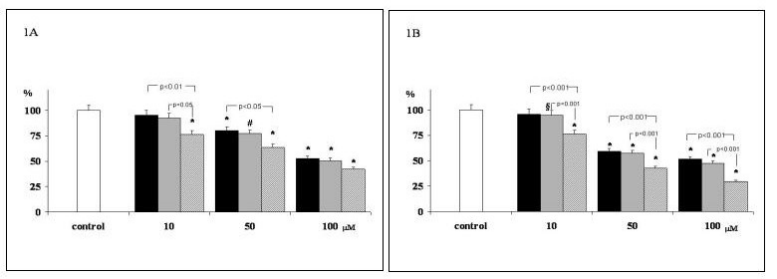
Normalized GAPDH gene expression data in PC-3 (Fig.1A) and in DU-145 (Fig.1B) prostate cancer cells. Control was used as calibrator. Fig.1A: In PC-3 and DU-145 cells amino-bisphosphonates ( Pamidronate,  Alendronate,  Zoledronate) lowered in a dose-dependent manner the GAPDH gene expression. * p < 0.001 vs control; # p < 0.005 vs control; § p < 0.05 vs control.

In PC-3 cells at 10 μM, the lowest dose tested, only Zoledronate significantly lowered the gene expression with respect to control (p < 0.001), whereas Alendronate and Pamidronate significantly down-regulate GAPDH gene expression at a concentration of 50 (p < 0.001 and p < 0.005, respectively) and 100 μM (p < 0.001). Comparing the effects among the different BPs, Zoledronate induced a greater inhibition of GAPDH gene expression at concentrations of 10 μM with respect to the Pamidronate (p < 0.01) and Alendronate (p < 0.05). Zoledronate lowered significantly the GAPDH gene expression at a concentration of 50 μM with respect to the Pamidronate (p < 0.05). At a concentration of 100 μM, amino-bisphosphonates showed a similar inhibition of GAPDH gene expression (Figure 1A).

Alendronate and Zoledronate induced a significant down-regulation at 10 μM with respect to control (p < 0.05 and p < 0.001, respectively) in DU-145 cell line. At the doses of 50 and 100 μM all the amino-BPs tested significantly lowered GAPDH gene expression with respect to control (p < 0.001). Zoledronate at concentrations of 10, 50 and 100 μM significantly down-regulated GAPDH gene expression with respect to Pamidronate and Alendronate at the same doses (p < 0.001). (Figure 1B).

**Figure 2:**
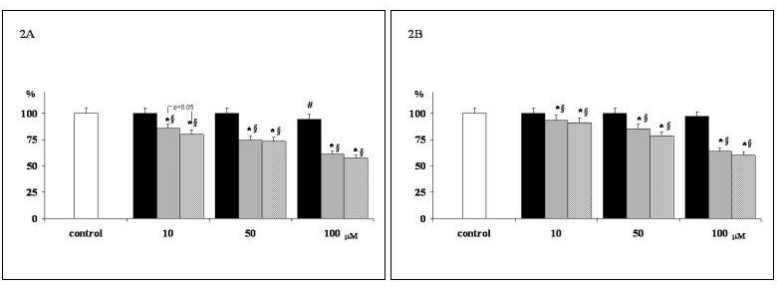
Normalized GAPDH gene expression data in T-47D (Fig.2A) and MCF-7 (Fig.2B) breast cancer cells. Control was used as calibrator. Amino-BPs ( Alendronate,  Pamidronate) significantly down-regulated the GAPDH expression with respect to control in a dose-dependent manner, whereas Non-Amino-BPs ( Clodronate) down-regulated this expression at 100 μM only in T-47D breast cancer cell line. * p < 0.001 vs control; # p < 0.05 vs control; § p < 0.001 vs Clodronate

In the T-47D cells Alendronate and Pamidronate significantly lowered GAPDH gene expression with respect to control in a dose dependent manner with all the doses tested (p < 0.001), whereas Clodronate down-regulated expression only at 100 μM (p < 0.05) (Figure 2A). However, Alendronate and Pamidronate at 10, 50 and 100 μM significantly decreased GAPDH gene expression with respect to all the doses of Clodronate (p < 0.001). At 10 μM Alendronate significantly down-regulated the gene expression with respect to Pamidronate at the corresponding concentration (p < 0.05).

We found similar results in MCF-7 cells. With all the doses Alendronate and Pamidronate significantly down-regulated GAPDH gene expression with respect to control in a dose dependent manner (p < 0.001), whereas Clodronate decreased, even if not significantly, this expression only at 100 μM. Alendronate and Pamidronate significantly down-regulated the GAPDH gene expression with respect to Clodronate at the same concentrations (p < 0.001) (Figure 2B).

## Discussion

In the present study, we have quantified by real time RT PCR the effect of the BPs on the expression of the GAPDH gene in prostate cancer cells (PC-3; DU-145) and in breast cancer cells (MCF-7; T-47D). Our results show a significant dose-dependent down-regulation of GAPDH gene expression after treatment of different cancer cell lines with different amino-BPs. Our results also indicate that Zoledronate was the most powerful bisphosphonate, whereas Clodronate, a non-amino bisphosphonate, exerted significant effect on GAPDH gene expression only at the highest concentration tested. This is, to our knowledge, the first report on the effects of BPs on GAPDH gene expression.

Some studies using GAPDH as a housekeeping gene showed the up-regulation of the expression of collagenase 3 [12], sialoprotein [26], TNF-α [27] TNF-α Converting Enzyme [28], osteoprotegerin [22], ALP and OC genes [29], by BPs. On the other hand, RANKL [30,31], PTHrp [32], osteopontin [33], Calcitonin receptor [29] were down-regulated. We may speculate that there could be two main reasons for the wide use of GAPDH gene as a housekeeping gene. The first being a general agreement that BPs act almost exclusively through the inhibition of mevalonate pathway, as confirmed by the evidence that GGOH and FOH, final products of mevalonate pathway, can completely reverse BPs effects [34]. However, some authors have documented effects independent from mevalonate pathway inhibition [35]. The second reason is that the large use of semiquantitative RT-PCR obscures the quantitative effects, such as the action of BPs on the expression of a single gene (also in the case of a control gene).

Our original observation has some interesting implications. First of all, as GAPDH mRNA expression is down-regulated in a dose-dependent manner by amino-BPs and we could speculate that every time a dose-dependent gene up-regulation is reported, it could be the effect of the concomitant down-regulation of the control gene expression, or it could be, at least, overestimated. On the contrary, the down-regulation of the housekeeping gene could obscure the detection of a possible specific gene down-regulation. A further consideration is that GAPDH could be considered as a novel target gene for BPs, showing not only a sensitive down-regulation but also reflecting the well accepted rank of relative in vitro and in vivo potency between amino and non-amino BPs and within amino-BPs [21].

It was shown that GAPDH is able to constitute a ternary complex with DNA and saframycin-related compound that induces a toxic response in cells. The demonstration that a specific binding interaction occurs between GAPDH, duplex DNA, and several members of the saframycin class of antiproliferative agents suggests GAPDH as a potential target for chemotherapeutic intervention [36]. GAPDH has a complex and evolving role in the nucleus, where it seems to act as a monomer [37]. GAPDH has been identified as a component of a multiprotein nuclear complex that recognized oligonucleotides incorporating the antileukemic agent thioguanosine [38], although GAPDH has not been shown to bind directly to the thioguanosine-modified DNA. Monomeric GAPDH has also been found as the main component of a multiprotein nuclear complex involved in transcriptional co-activation of the histone 2B promoter, required for S phase progression in the cell cycle [39].

These data raise the possibility that, at least in part, the direct effect of amino-BPs on cancer cells could be mediated by GAPDH down-regulation. Interestingly, by utilizing real time PCR, GAPDH mRNA has been down-regulated by many antineoplastic drugs [36,40] and it has been related to apoptosis [41].

This study was designed to investigate the possible effects of BPs on GAPDH mRNA expression, and to explore the reliability of GAPDH as a housekeeping gene in real time RT-PCR analysis. Therefore, the data obtained do not allow the definition of a specific mechanism of action of amino-BPs through GAPDH down-regulation and we can only speculate that BPs could affect vitality in cancer cells in other ways apart from through the inhibition of the mevalonate pathway, i.e. through the down-regulation of GAPDH.

## Conclusion

The use of GAPDH as a control gene, in particular in studies investigating the effects of BPs on bone or cancer cells, seems to be inappropriate and RT-PCR data on the effects of BPs in cancer cells should be reviewed by a quantitative approach using real time PCR, with a different housekeeping gene.

## Competing interests

The authors of this manuscript dclare that they have no financial, political, religious, academic, intellectual, commercial or any other competing interests.

## Authors' contributions

MTV participated in the design of the study and carried out the molecular assays

FB participated in the design of the study and contributed to draft the manuscript

LDC participated in the design of the study and performed the statistical analysis

GA helped to perform the statistical analysis and participated to draft the manuscript

SZ carried out the molecular assays

MZ carried out the molecular assays

EB carried out the molecular assays and drafted the manuscript

OV participated in the design of the study and contributed to draft the manuscript

VLC conceived the study and coordinated to draft the manuscript

All authors read and approved the final manuscript.

## Pre-publication history

The pre-publication history for this paper can be accessed here:
